# Real-Time Polymerase Chain Reaction in Detecting Active Hepatitis C Virus Infection: A Retrospective Analysis

**DOI:** 10.7759/cureus.104533

**Published:** 2026-03-02

**Authors:** Purvaja Muthukumarasamy, Priyadharshini Elumalai, Krithika Gopalakrishnan, Shanthi Mariappan, Sudhabharathi Reju

**Affiliations:** 1 Department of Microbiology, Sri Ramachandra Institute of Higher Education and Research, Chennai, IND

**Keywords:** communicable disease, global health, hcv rna, hepatitis c virus, liver enzymes, viral load

## Abstract

Introduction

Hepatitis C virus (HCV) infection is a major worldwide health concern. It is a major cause of cirrhosis, hepatocellular carcinoma, and chronic hepatitis. Testing for anti-HCV antibodies is common for screening purposes, but it does not distinguish between an active and a past infection. A real-time polymerase chain reaction (RT-PCR)-based detection of HCV ribonucleic acid (RNA) is used to confirm active infection following initial serological screening. The current study aimed to assess the proportion of HCV RNA positivity, the distribution of viral load, and its association with demographic, serological, and biochemical markers in patients screened for HCV infection.

Materials and methods

This study was a retrospective analysis carried out at a tertiary care hospital in Chennai, India. It involved 383 patients who were screened for hepatitis C virus infection between July 2023 and July 2024. HCV RNA detection and viral load quantification were performed using RT-PCR. Testing for anti-HCV antibodies was carried out using a chemiluminescent microparticle immunoassay (CMIA). The liver function parameters were obtained from laboratory records at the time of HCV testing. The clinical details of the study patients were assessed retrospectively from the medical records. Demographic details such as age, gender, and clinical diagnosis were collected. The results were tabulated, and statistical analysis was performed.

Results

Of the 383 individuals tested, 98 (25.6%) were positive for HCV RNA. Individuals aged ≥60 years constituted 56.6% of cases within that age group and accounted for the majority of HCV RNA-positive individuals, demonstrating a statistically significant association with HCV RNA positivity (chi-square {χ²} = 88.37; p < 0.001). Women demonstrated a higher HCV RNA positivity rate (30.0%) compared to men (23.6%), though this difference was not statistically significant (p = 0.181). Viral load analysis showed that 51.0% of individuals had low-level viremia (11-100,000 IU/mL), while 26.5% had viral loads exceeding 500,000 IU/mL. Liver enzyme elevation did not differ significantly between dialysis and non-dialysis groups (p = 0.586). However, a significant association was observed between higher viral load (>500,000 IU/mL) and liver enzyme elevation (p = 0.030). Among HCV RNA-positive individuals, 29.6% were nonreactive for anti-HCV antibodies.

Conclusion

This study focuses on the importance of molecular testing for detecting active HCV infections in tertiary care settings. Compared to conventional screening methods, HCV RNA testing provides a more reliable confirmation of active disease.

## Introduction

Hepatitis C virus (HCV) is a single-stranded RNA virus belonging to the family Flaviviridaeand the genus *Hepacivirus.* It represents a major global health concern as a leading cause of chronic blood-borne infection [1,2]. Direct percutaneous exposure to blood is a primary mode of HCV transmission. Other routes include injection drug use, the transfusion of infected blood products, hemodialysis-related exposure, and occupational exposure among healthcare workers. Less frequent routes include sexual and vertical transmission. However, in many cases, the source remains unidentified. Most infected individuals develop chronic hepatitis, which can progress to cirrhosis [3].

Diagnostic methods include enzyme immunoassays, which detect antibodies to HCV. However, these tests cannot differentiate between active and past infection. Nucleic acid-based assays, such as real-time polymerase chain reaction (RT-PCR), are considered the gold standard for HCV detection because they identify circulating hepatitis C virus ribonucleic acid (HCV RNA). These assays are useful for the early diagnosis of acute infection following exposure and for monitoring patients receiving direct-acting antiviral (DAA) therapy [3].

According to the World Health Organization Global Hepatitis Report 2024, an estimated 50 million individuals were living with chronic HCV infection globally in 2022. India accounts for a considerable proportion of this burden, with approximately 5.5 million people affected, making it the country with the second-highest viral hepatitis burden worldwide after China [4]. The World Health Organization aims to eliminate viral hepatitis as a public health threat by 2030, targeting a 90% reduction in new infections and a 65% reduction in hepatitis-related mortality compared to 2015 levels [5]. In India, the National Viral Hepatitis Control Program (NVHCP) was launched in 2018 to strengthen nationwide screening, confirmatory molecular testing, and free access to treatment for viral hepatitis, including hepatitis C [6]. Overall, India contributed 11.6% of the global viral hepatitis burden in 2022, indicating the need for strengthened diagnostic and screening strategies to identify active infection [5].

This retrospective study assessed the proportion of HCV RNA positivity and its association with demographic, serological, and biochemical markers, as well as viral load distribution among patients screened for HCV infection at a tertiary care center.

## Materials and methods

This study was a retrospective analysis carried out at a tertiary care hospital in Chennai, India, involving 383 patients who were screened for Hepatitis C virus infection between July 2023 and July 2024. The study was approved by the Institutional Ethics Committee (IEC) of Sri Ramachandra Institute of Higher Education and Research (approval number: CSP-MED/25/AUG/120/235).

Anti-HCV antibodies

The anti-HCV antibody detection from serum samples was tested using chemiluminescent microparticle immunoassay (CMIA) for the qualitative detection of anti-HCV antibodies (Abbott, Nyon, Switzerland). It uses the HCr43 protein, which is composed of two noncontiguous coding segments of the HCV genome (33c and core and c100 3 {putative nonstructural NS3 and NS4}). According to the analyzer, a result exceeding 1.0 signifies a reactive sample, indicating the potential presence of HCV antibodies.

RT-PCR for HCV detection and viral load

Hepatitis C virus ribonucleic acid (HCV RNA) was extracted from plasma using the QIAamp Viral RNA Mini Kit (QIAGEN, Hilden, Germany), in accordance with the manufacturer's instructions. The extracted RNA was stored at -80°C until further processing. Amplification was performed using the AltoStar HCV RNA PCR amplification kit (Altona, Hamburg-Altona, Germany), which utilizes real-time one-step polymerase chain reaction (PCR) technology. The analytical detection limit is 11.1 IU/mL for the AltoStar HCV RT-PCR Kit. Real-time PCR was performed using the Rotor-Gene Q (RGQ) real-time PCR analyzer (QIAGEN, Hilden, Germany).

Liver function tests

The liver function parameters, namely, aspartate aminotransferase (AST), alanine aminotransferase (ALT), and alkaline phosphatase (ALP), were obtained from laboratory records at the time of HCV testing.

The clinical details of the study patients were perused retrospectively from the medical records. Demographic details such as age, gender, and clinical diagnosis were collected.

Statistical analysis

Statistical analysis was performed using IBM SPSS Statistics version 26 (IBM Corp., Armonk, NY). Pearson's chi-square (χ²) test was used to assess associations between categorical variables, including age, gender, HCV RNA status, dialysis status, and liver enzyme elevation. Fisher's exact test was applied for the comparison of liver enzyme status with viral load categories due to smaller subgroup frequencies. A p-value of <0.05 was considered statistically significant. As this was a retrospective laboratory-based study including all eligible patients during the study period, no prior sample size calculation was performed.

## Results

Distribution of HCV RNA status in the study population (n = 383)

**Figure 1:**
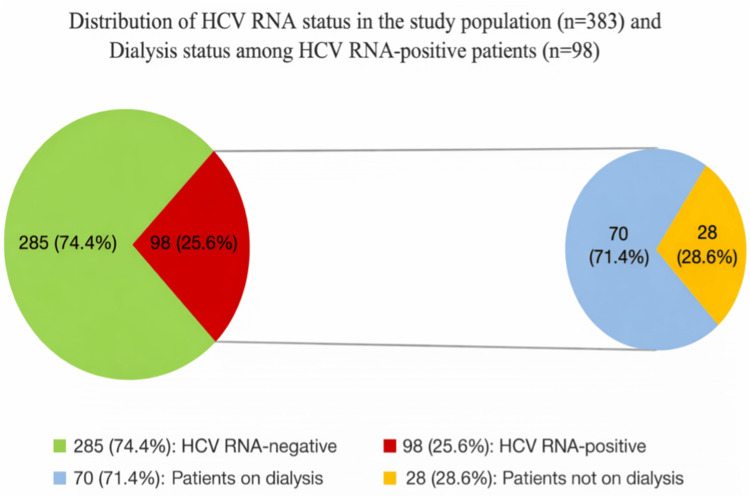
Distribution of HCV RNA status in the study population (n = 383) and dialysis status among HCV RNA-positive patients (n = 98) Green: HCV RNA-negative (n = 285, 74.4%). Red: HCV RNA-positive (n = 98, 25.6%). Blue: patients on dialysis among HCV RNA-positive (n = 70, 71.4%). Yellow: patients not on dialysis among HCV RNA-positive (n = 28, 28.6%) HCV RNA: hepatitis C virus ribonucleic acid

A total of 383 patients were screened for HCV infection. Among these, 98 patients (25.6%) were HCV RNA-positive, while 285 patients (74.4%) were HCV RNA-negative. Among the HCV RNA-positive patients (n = 98), the majority (n = 70, 71.4%) were on dialysis, whereas 28 patients (28.6%) were not on dialysis (Figure 1).

Association between age and HCV RNA status

HCV RNA positivity was significantly higher among patients aged ≥60 years (56.6%) compared to those aged <60 years (11.2%) (χ² = 88.37; p < 0.001). In contrast, most HCV RNA-negative individuals (88.8%) belonged to the <60-year age group (Table 1).

**Table 1:** Association between age and HCV RNA status Percentages in parentheses. Pearson's chi-square (χ²) test was used to assess the association between gender and HCV RNA status. A p-value of <0.05 was considered statistically significant HCV RNA: hepatitis C virus ribonucleic acid

Age Group (Years)	HCV RNA-Positive (n = 98)	HCV RNA-Negative (n = 285)	χ² Value	P-value
<60	29 (11.2%)	232 (88.8%)	88.37	p < 0.001
≥60	69 (56.6%)	53 (43.4%)		

Association between gender and HCV RNA status

Among men (n = 263), 62 (23.6%) were HCV RNA-positive, whereas among women (n = 120), 36 (30%) were HCV RNA-positive. Chi-square analysis showed no statistically significant association between gender and HCV RNA positivity (χ² = 1.79; p = 0.181) (Table 2).

**Table 2:** Association between gender and HCV RNA status Percentages in parentheses. Pearson's chi-square (χ²) test was used to assess the association between gender and HCV RNA status. A p-value of <0.05 was considered statistically significant HCV RNA: hepatitis C virus ribonucleic acid

Gender	HCV RNA-Positive (n = 98)	HCV RNA-Negative (n = 285)	χ² Value	P-value
Male (n = 263)	62 (23.6%)	201 (76.4%)	1.79	0.181
Female (n = 120)	36 (30.0%)	84 (70.0%)		

Distribution of viral load among HCV RNA-positive patients (n = 98)

**Figure 2:**
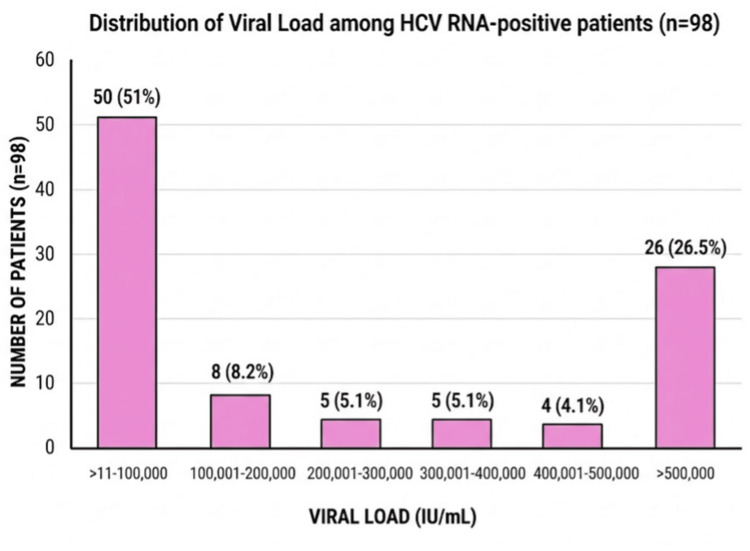
Distribution of viral load among HCV RNA-positive patients (n=98) Values are expressed as number (percentage). Percentages were calculated using the total number of HCV RNA-positive patients (n = 98) as the denominator. Viral load is expressed in international units per milliliter (IU/mL) HCV RNA: hepatitis C virus ribonucleic acid

Among HCV RNA-positive patients, 51% had viral loads between 11 and 100,000 IU/mL, and 26.5% had viral loads of >500,000 IU/mL (Figure 2).

Association between liver enzyme elevation and dialysis status among HCV RNA-positive patients

A chi-square test of independence was performed to examine the association between dialysis status and liver enzyme elevation. The proportion of patients with elevated liver enzymes was comparable between the dialysis group (77.1%) and the non-dialysis group (82.1%). These findings indicate that dialysis status was not significantly associated with liver enzyme abnormalities (χ² = 0.30; p = 0.586) (Table 3).

**Table 3:** Association between liver enzyme elevation and dialysis status among HCV RNA-positive patients Percentages in parentheses. Liver enzymes include serum aspartate aminotransferase (AST), alanine aminotransferase (ALT), and alkaline phosphatase (ALP). "Enzymes Elevated (Any)" indicates the elevation of at least one of the measured liver enzymes above the laboratory reference range. Pearson's chi-square (χ²) test was used to assess the association between liver enzyme elevation and dialysis status. A p-value of <0.05 was considered statistically significant HCV RNA: hepatitis C virus ribonucleic acid

Dialysis Status	Enzymes Elevated (Any)	Enzymes Normal	χ² Value	P-value
Dialysis group (n = 70)	54 (77.1%)	16 (22.9%)	0.30	0.586
Non-dialysis group (n = 28)	23 (82.1%)	5 (17.9%)		

Association between liver enzyme elevation and viral load among HCV RNA-positive patients

Patients with viral loads of >500,000 IU/mL demonstrated a significantly higher proportion of enzyme elevation (95.8%) compared to those with viral loads of <500,000 IU/mL (73%) (Fisher's exact test, p = 0.030). Normal enzyme levels were more frequently observed in the lower-viral-load group (27%) (Table 4).

**Table 4:** Association between liver enzyme elevation and viral load among HCV RNA-positive patients Percentages in parentheses. Liver enzymes include serum aspartate aminotransferase (AST), alanine aminotransferase (ALT), and alkaline phosphatase (ALP). "Enzymes Elevated" indicates the elevation of at least one of the measured liver enzymes above the laboratory reference range. Fisher's exact test was applied due to small expected cell counts. A p-value of <0.05 was considered statistically significant HCV RNA: hepatitis C virus ribonucleic acid

Viral Loads (IU/mL)	Enzymes Elevated (Any)	Enzymes Normal	Fisher's Exact P-value
<500,000 (n = 74)	54 (73%)	20 (27%)	0.030
>500,000 (n = 24)	23 (95.8%)	1 (4.2%)	

Distribution of HCV viral load among anti-HCV antibody-negative, HCV RNA-positive patients

**Figure 3:**
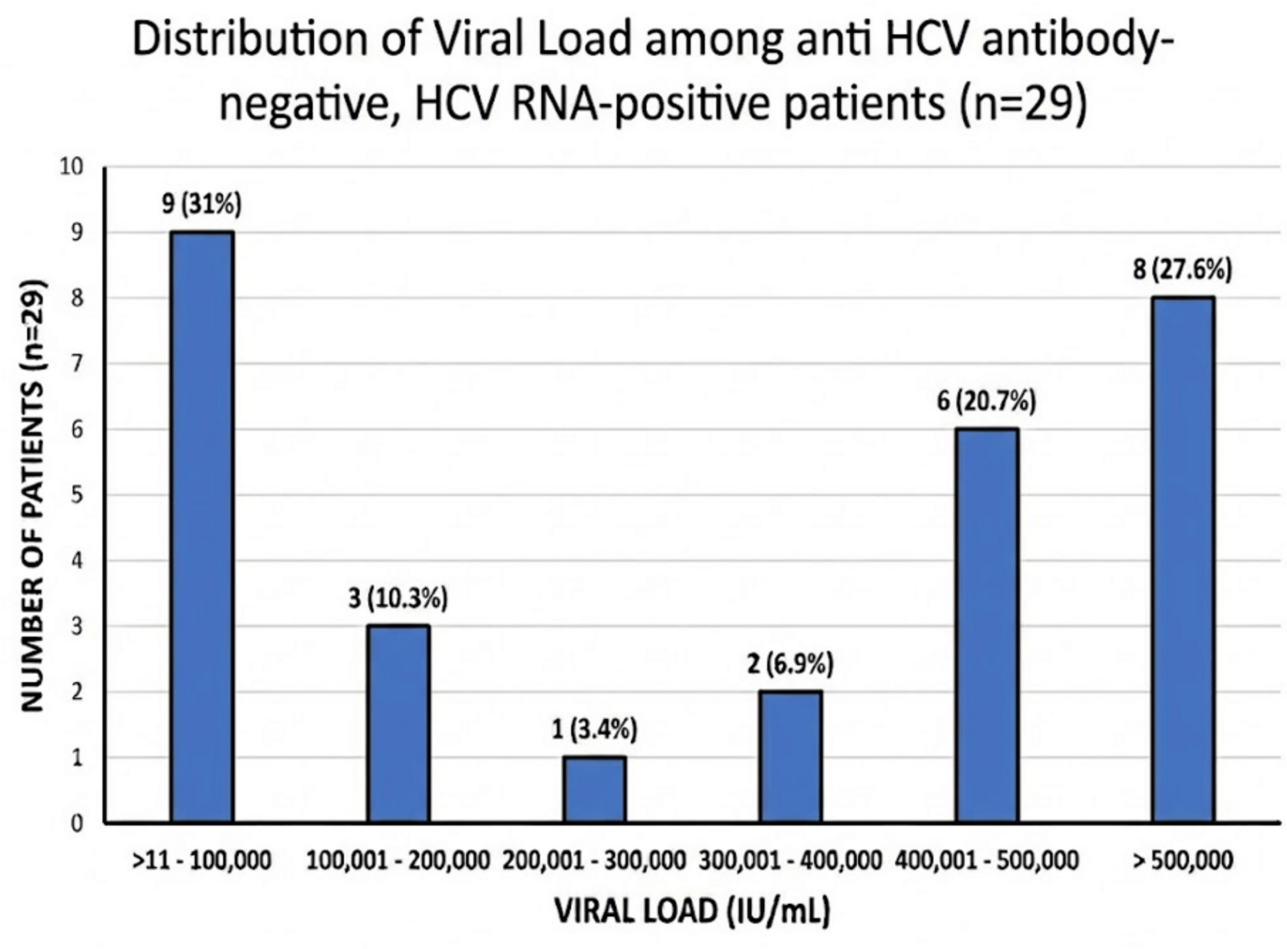
Distribution of HCV viral load among anti-HCV antibody-negative, HCV RNA-positive patients A bar graph where the X-axis represents viral load categories (IU/mL) and the Y-axis represents the number of patients (n = 29). Percentages displayed above each bar indicate the proportion within the antibody-negative group. Viral load is measured in international units per milliliter (IU/mL) HCV RNA: hepatitis C virus ribonucleic acid

Figure 3 depicts the distribution of viral load levels among anti-HCV antibody-negative, HCV RNA-positive patients (n = 29). Viral loads between 11 and 100,000 IU/mL were observed in 31% of the patients, while 48.3% demonstrated viral loads exceeding 400,000 IU/mL.

## Discussion

HCV has the ability to persist in the liver and establish chronic infection while remaining latent for years. HCV often remains undetected until significant hepatic damage has occurred. This silent progression makes the identification of active viremia through molecular testing a critical step in preventing long-term complications.

A major concern in HCV transmission is the risk of healthcare-associated transmission. Adherence to strict standard precautions in dialysis units, including the use of dedicated dialysis machines for infected patients, helps reduce cross-contamination and the risk of nosocomial spread [2,7].

In our study, HCV RNA positivity was observed in 98 (25.6%) of the patients. This finding is comparable to studies from Turkey (20.7%). Higher rates have been reported from South Korea (57%) and China (34.1%), while studies from Indonesia show a wide variability range (3.7%-56.3%) [8-10]. These findings are consistent with Indian studies, where HCV prevalence in dialysis units has ranged from 10% to 40%. A study from West Bengal reported a viremia rate of 50.64% among seroreactive patients [11]. Studies from Kerala and Andhra Pradesh documented lower RNA positivity rates of 8%-10% [2]. Data from Western Uttar Pradesh showed a prevalence of 9.33% among patients on maintenance hemodialysis, reflecting notable regional variation across India [12].

HCV RNA positivity was significantly higher among patients aged ≥60 years (56.6%) compared to those aged <60 years (11.2%). In a study conducted by Maheta et al., the highest burden of infection was concentrated in the 41-60 age group, accounting for 41.55% [13]. In contrast, the positivity rate among individuals aged >60 years was considerably lower (15%) [13,14].

Women had a slightly higher (30%) HCV RNA positivity rate than men (23.6%), but this difference was not statistically significant (p = 0.181). Large epidemiological studies often find similar or slightly higher HCV seroprevalence in women, whereas active viremia and progression to fibrosis and cirrhosis are frequently more pronounced in men, possibly due to sex hormone-related immunomodulation and differential exposure risks such as injection drug abuse [15].

Viral loads displayed a distinctly bimodal pattern, with over half of HCV RNA-positive patients (50, 51%) concentrated in the lowest category of viremia (11-100,000 IU/mL), while a sizeable high-viremia subgroup (26, 26.5%) exceeded 500,000 IU/mL, and comparatively, fewer patients fell within the ranges of 1-500,000 IU/mL.

Clinically, low-to-moderate viral loads are associated with a relatively favorable prognosis and excellent response to pan-genotypic DAAs, which now achieve sustained virologic response rates above 95% across age groups. High baseline viral loads, although less influential in the DAA era, remain relevant as they are linked to faster fibrosis progression and reduced spontaneous clearance. Therefore, viral load estimation is essential to support the initiation of early treatment even in patients with preserved liver function [16].

In the present study, liver enzyme elevation was observed in 77.1% of HCV RNA-positive patients undergoing dialysis and in 82.1% of non-dialysis patients, with no statistically significant difference (p = 0.586). This comparable prevalence suggests that dialysis status alone did not independently influence liver enzyme elevation. The high frequency of enzyme elevation in both groups indicates that hepatic injury in HCV-infected patients is more likely related to viral and host factors than to dialysis-associated exposures [17].

Patients exhibiting high viral loads (>500,000 IU/mL) showed a significantly higher frequency of enzyme elevation (95.8%) compared to those with lower viral loads (73%) (p = 0.030). This finding suggests that higher levels of viral replication may contribute more directly to hepatocellular injury and inflammatory activity [18].

Among the HCV RNA-positive patients, 69 (70.5%) were also anti-HCV antibody-reactive. Nevertheless, 29 (29.6%) of the patients with detectable HCV RNA were antibody-negative. This may reflect the serological window period of acute infection, where HCV RNA is detectable within 1-2 weeks of infection, and seroconversion can be delayed for months or may never occur, as seen in dialysis patients and transplant recipients, where delayed or blunted antibody responses may occur. This discordance, in which nearly one-third of the patients with viremia tested negative for antibodies, highlights a limitation of serological screening in individuals undergoing hemodialysis, as the occult infections are mainly linked to immune dysfunction related to uremia, which disrupts the cooperation between T-cells and B-cells. As a result, there is an inability to generate anti-HCV antibodies, even in the presence of active viral replication [18,19].

In contrast, anti-HCV serology can overestimate active disease burden, given the long-term persistence of antibodies following spontaneous or treatment-induced clearance and being falsely positive in low-prevalence settings due to cross-reactivity. Current nephrology guidelines recommend nucleic acid testing for diagnosing active infection, guiding treatment decisions, and monitoring response, particularly in dialysis populations [20,21]. The bimodal viral load distribution within the antibody-positive subset in this study, with clustering at very low and very high viremia, further illustrates the limited value of antibody status in predicting replication intensity or progression risk and reinforces the need for confirmatory HCV RNA testing to ensure that patients with ongoing viremia are accurately identified and treated [22].

The presence of anti-HCV antibodies does not distinguish between active and past infections. They can be observed in the bloodstream about 7-8 weeks after exposure, and HCV RNA testing can point to an infection as early as one week after exposure. Patients who have tested positive for anti-HCV antibodies are advised to undergo testing for HCV RNA as well, and those who have had HCV RNA levels that were previously undetectable, whether due to spontaneous resolution or antiviral treatment, should have regular follow-up assessments.

In individuals receiving hemodialysis, regular screening for anti-HCV antibodies and the periodic monitoring of liver enzymes are recommended. If unexplained elevations in ALT levels are observed, follow-up HCV RNA testing should be performed rather than relying solely on serological tests [23].

Limitations

The retrospective design of this single-center study limits the generalizability of the findings. Additionally, the absence of longitudinal follow-up restricted the assessment of temporal changes in viral load. Furthermore, the limited availability of detailed clinical parameters constrained comprehensive clinical correlation.

## Conclusions

This retrospective study identified a substantial proportion of HCV RNA positivity among individuals screened in a tertiary care setting. This included a significant subset of patients with viremia who were nonreactive for anti-HCV antibodies. The findings showed that RT-PCR is important for confirming active HCV infection and that serological testing alone may not be sufficient. These observations support the incorporation of molecular testing into HCV screening approaches, particularly for high-risk groups such as patients undergoing hemodialysis.
